# A Rapid Method for Characterization of Protein Relatedness Using Feature Vectors

**DOI:** 10.1371/journal.pone.0009550

**Published:** 2010-03-05

**Authors:** Kareem Carr, Eleanor Murray, Ebenezer Armah, Rong L. He, Stephen S.-T. Yau

**Affiliations:** 1 Department of Mathematics, Statistics and Computer Science, University of Illinois at Chicago, Chicago, Illinois, United States of America; 2 Department of Epidemiology, Mailman School of Public Health, Columbia University, New York, New York, United States of America; 3 Department of Biological Sciences, Chicago State University, Chicago, Illinois, United States of America; Virginia Tech, United States of America

## Abstract

We propose a feature vector approach to characterize the variation in large data sets of biological sequences. Each candidate sequence produces a single feature vector constructed with the number and location of amino acids or nucleic acids in the sequence. The feature vector characterizes the distance between the actual sequence and a model of a theoretical sequence based on the binomial and uniform distributions. This method is distinctive in that it does not rely on sequence alignment for determining protein relatedness, allowing the user to visualize the relationships within a set of proteins without making *a priori* assumptions about those proteins. We apply our method to two large families of proteins: protein kinase C, and globins, including hemoglobins and myoglobins. We interpret the high-dimensional feature vectors using principal components analysis and agglomerative hierarchical clustering. We find that the feature vector retains much of the information about the original sequence. By using principal component analysis to extract information from collections of feature vectors, we are able to quickly identify the nature of variation in a collection of proteins. Where collections are phylogenetically or functionally related, this is easily detected. Hierarchical agglomerative clustering provides a means of constructing cladograms from the feature vector output.

## Introduction

Recent advances in biotechnology have allowed sequencing of millions of proteins from a wide spectrum of organisms and this information is rapidly becoming accessible to any researcher with an internet connection. For instance, the UniProtKB database currently contains over 7 million protein sequences and is updated every three weeks [1]. Current protein alignment methods are often slow and require assumptions about relatedness and evolutionary mechanisms [2]. In order to make use of the vast amount of protein data available, a method for quickly delineating large numbers of proteins into related types is necessary. As a solution, we propose a method for quantifying the frequency and position of amino acids within a protein, and demonstrate the ease, rapidity and usefulness of this technique for uncovering phylogenetic and functional relationships within protein families, using protein kinase C (PKC), hemoglobin and myoglobin as examples.

One of us (S.Y.) previously designed three types of parameters for use in clustering amino acid sequences [3]. Here, we reconceptualize these parameters, making them more statistical in character and more applicable to measuring protein similarity. The new parameters measure the degree to which the distribution of amino acids in a particular protein deviates from a theoretical protein containing an equal number of residues but undergoing neutral evolution. This measure allows us not only to characterize the extent to which the distribution of amino acids in a protein deviates from the expected but also the distance between proteins. Our theoretical model of the distribution of amino acids in a protein assumes that for any given amino acid, the locations of residues of that amino acid are distributed uniformly and the number of residues is distributed binomially.

In contrast to previous methods of constructing distance measures to determine protein relatedness [4], our method does not require performing multiple sequence alignment. Instead, this method creates measures of protein relatedness based on the distribution of amino acids in the proteins. Differences in these measures are then used to determine the difference between two proteins. In doing so, this method requires no assumptions about the way in which certain amino acids may be inserted or deleted. This allows us to look at protein difference in an abstract way, without making assumptions about the mechanisms by which these differences may arise.

## Results

### Implementation of the Feature Vectors

**Figure 1:**
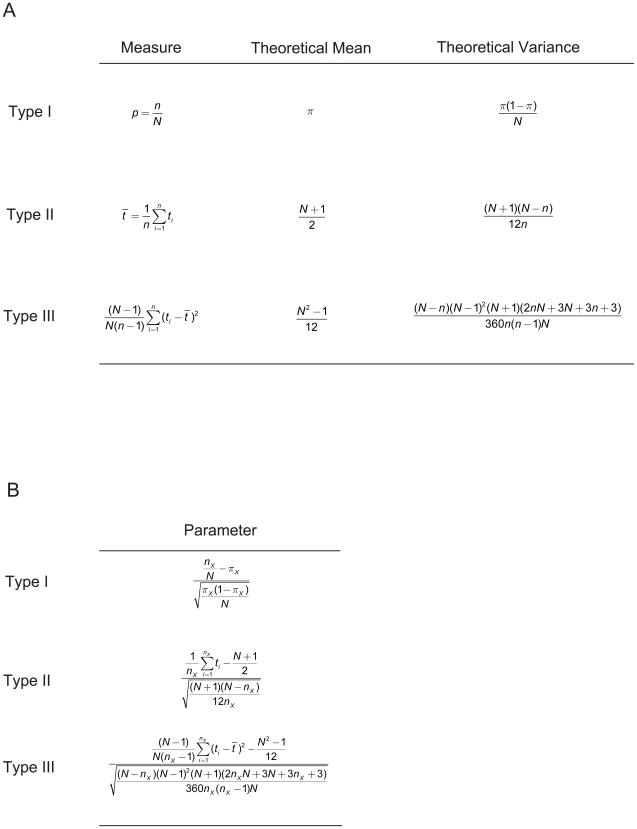
Calculation of the Protein Feature Vectors. (A) Intermediate calculations necessary to compute three component parameters of the feature vector, parameters are computed as (measure − mean)/sqrt(variance); (B) sample parameters of Types I–III for an amino acid of type X, the full 60-dimentional feature vector for a given protein will include parameters for all amino acid types in that protein (3 parameters for each of 20 amino acid types).

The selection pressure to which proteins are subjected affects both the number of residues in the protein [5] and the identity and location of these residues. By using a feature vector, which is an ordered list of numbers that characterize the distribution of amino acids in a protein, we can describe this selection pressure. Our method uses a feature vector constructed with three types of parameters (Fig. 1). The three types of parameters can be described as compositional, centrodial and distributional. Compositional parameters (type I) measure the extent to which the proportion of amino acids deviate from the expected. Centroidial parameters (type II) measure the extent to which amino acids tend to be in a particular region of the protein. Distributional parameters (type III) measure the extent to which amino acids cluster along the length of the protein. All parameters are adjusted for the length of the protein. From simulation of parameter distributions, it can be seen that, for some range of protein lengths and frequencies of amino acids, individual parameters have an approximately Gaussian distribution over large data sets (data not shown). The distance between feature vectors is measured as Euclidean distance.

To describe any protein using the feature vector method, we must first compute parameters of type I, II and III for each of the twenty amino acids which occur in proteins (Supplement S1). The parameter for each amino acid can be computed based on the information in figure 1A, by subtracting the theoretical mean from the measure and dividing by the square root of the theoretical variance. As an example, the formula for a parameter of type I for glycine (G) is:
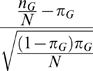



Here, *π_G_* is the probability of glycine occurring in a theoretical dataset of proteins under neutral evolution. This probability is set by the user and can vary for a given dataset. For the current analyses, we use by the number of genetic codons for glycine divided by 64. Alternatively, one can set *π_G_* to the frequency of glycine in the total dataset of proteins. The variable *n_G_* is the number of glycines in the protein and *N* is the length of the protein.

The following 9 steps detail the specifics of computing the parameters for the feature vector method and of using the feature vectors to categorize and analyze proteins. C++ and Mathematica (Wolfram Research, Urbana-Champaign, IL) code for steps 1–8 is available in the supplementary online materials (Sample Code S1 and S2). Step 9, performing a principle component analysis (PCA), can be done using Matlab software (Mathworks, Natwich, MA) or similar statistical software. Matlab code is provided in the supplementary online materials (Sample Code S3).

1. Count the number of amino acids of each type and note the length of the protein. We denote the length of protein as N and the number of amino acids of each type as n_A_, n_R_, n_N_, n_D,_ n_C_, n_E_, n_Q_, n_G,_ n_H_, n_I_, n_L_, n_K,_ n_M_, n_F,_ n_P_, n_S,_ n_T_, n_W,_ n_Y_ and n_V_. Use these numbers to compute the proportion of amino acids in the protein, the type I measure (fig. 1A): p_A_, p_R_, p_N_, p_D,_ p_C_, p_E_, p_Q_, p_G,_ p_H_, p_I_, p_L_, p_K,_ p_M_, p_F,_ p_P_, p_S,_ p_T_, p_W,_ p_Y_ and p_V_. (Where, A = Alanine, R = Arginine, N = Asparagine, D = Aspartic acid, C = Cysteine, E = Glutamic, Q = Glutamine, G = Glycine, H = Histidine, I = Isoleucine, L = Leucine, K = Lysine, M = Methionine, F = Phenylalanine, P = Proline, S = Serine, T = Threonine, W = Tryptophan, Y = Tyrosine, V = Valine).

2. For each amino acid, find the indices of the positions in the protein sequence which contain that amino acid (we count the first position as one and not zero).

3. Use the indices from step 2 to compute the mean position of each amino acid, the type II measure: m_A_, m_R_, m_N_, m_D,_ m_C_, m_E_, m_Q_, m_G,_ m_H_, m_I_, m_L_, m_K,_ m_M_, m_F,_ m_P_, m_S,_ m_T_, m_W,_ m_Y_ and m_V_. The type II theoretical mean is calculated as the average of all possible positions in a protein of length N.

4. Using the indices from step 2 compute the unbiased variance, type III measure, of the set of indices of each amino acid: v_A_, v_R_, v_N_, v_D,_ v_C_, v_E_, v_Q_, v_G,_ v_H_, v_I_, v_L_, v_K,_ v_M_, v_F,_ v_P_, v_S,_ v_T_, v_W,_ v_Y_ and v_V._ The type III theoretical mean is calculated as half of one less than the square of the length.

5. The type I theoretical mean is the *a priori* estimate of the probability of the occurrence of each amino acid: π_A_, π_R_, π_N_, π_D,_ π_C_, π_E_, π_Q_, π_G,_ π_H_, π_I_, π_L_, π_K,_ π_M_, π_F,_ π_P_, π_S,_ π_T_, π_W,_ π_Y_ and π_V,_ This value was taken to be the number of codons corresponding to a particular amino acid divided by the total number of coding codons, although it could also more appropriately be taken as the rate of occurrence of amino acids in the population from which the sample proteins were selected. Use the type I mean to compute the type I theoretical variance of index positions given the observed number of amino acids.

6. Compute the type II theoretical variance in the mean and the type III theoretical variance of the variance given the observed number of amino acids as shown in figure 1A.

7. Normalize the measures computed in steps 1, 3 & 4 by subtracting the theoretical means, computed in steps 3, 4 & 5, and dividing by the square root of the theoretical variances, computed in steps 5 & 6.

8. Assemble the parameters into a 60 dimensional vector.

9. Using principle components analysis (PCA) or some other means of high dimensional data analysis, search for clusters or patterns in the protein data.

This method can be adapted for use in analyzing DNA or RNA sequences. To create a feature vector for a given DNA sequence, one need only create parameters of types I, II and III for each nucleic acid type, and then combine these parameters into a feature vector. The resulting DNA feature vector will be 12-dimensional, with 3 types of parameter for each of 4 types of nucleic acid, compared to the 60-dimensional protein feature vector (Tables S1, S2, and S3 contain feature vectors for our PKC, hemoglobin and myoglobin datasets, respectively).

To demonstrate the utility of this method, we applied our feature vector method to an exhaustive set of 128 proteins from the PKC family and a set of 904 hemoglobins and 150 myoglobins. The UniProt KB accession numbers for these proteins are provided in the supplementary online materials (see Tables 1–4 for accession numbers; see Data Set S1 for additional information).

**Table 1:** NCBI or SwissProt/UniProt Accession Numbers for Protein Kinase C dataset.

NP_001006133	P09215	P87253	Q5R4K9	Q7SY24	Q9UVJ5
NP_001008716	P09216	P90980	Q5TZD4	Q7SZH7	Q9Y792
NP_001012707	P09217	Q00078	Q62074	Q7SZH8	Q9Y7C1
O01715	P10102	Q02111	Q62101	Q7T2C5	XM_391874
O17874	P10829	Q02156	Q64617	Q86XJ6	XP_001066028
O19111	P10830	Q02956	Q69G16	Q86ZV2	XP_001116804
O42632	P13677	Q04759	Q6AZF7	Q873Y9	XP_001147999
O61224	P16054	Q05513	Q6BI27	Q8IUV5	XP_001250401
O61225	P17252	Q05655	Q6C292	Q8J213	XP_234108
O62567	P20444	Q15139	Q6DCJ8	Q8JFZ9	XP_421417
O62569	P23298	Q16974	Q6DUV1	Q8K1Y2	XP_540151
O76850	P24583	Q16975	Q6FJ43	Q8K2K8	XP_541432
O94806	P24723	Q19266	Q6GNZ7	Q8MXB6	XP_583587
O96942	P28867	Q25378	Q6P5Z2	Q8NE03	XP_602125
O96997	P34885	Q28EN9	Q6P748	Q90XF2	XP_683138
P04409	P36582	Q2NKI4	Q6UB96	Q91569	XP_849292
P05126	P36583	Q2U6A7	Q6UB97	Q91948	XP_851386
P05129	P41743	Q3UEA6	Q75BT0	Q99014	XP_851861
P05130	P43057	Q498G7	Q76G54	Q9BZL6	
P05696	P63318	Q4AED5	Q7LZQ8	Q9GSZ3	
P05771	P68403	Q4AED6	Q7LZQ9	Q9HF10	
P05772	P68404	Q4R4U2	Q7QCP8	Q9HGK8	

**Table 2 pone-0009550-t002:** SwissProt/UniProt Accession Numbers for Hemoglobin dataset – Part 1.

A1A4Q3	O24520	P01943	P01976	P02006	P02040	P02078	P02111	P02142	P07036	P08256
A1A4Q7	O24521	P01944	P01977	P02007	P02042	P02080	P02112	P02143	P07402	P08257
A1YZP4	O42425	P01945	P01978	P02008	P02044	P02081	P02113	P02208	P07403	P08258
A2TDC2	O76242	P01946	P01979	P02009	P02046	P02082	P02114	P02209	P07404	P08259
A2TDC3	O76243	P01947	P01980	P02010	P02047	P02083	P02115	P02213	P07405	P08260
A2V8C0	O77655	P01948	P01981	P02011	P02048	P02084	P02116	P04237	P07406	P08261
A2V8C1	O81941	P01949	P01982	P02012	P02049	P02085	P02117	P04238	P07407	P08422
A2V8C2	O88752	P01950	P01983	P02013	P02050	P02086	P02118	P04239	P07408	P08423
A4GTS5	O88754	P01951	P01984	P02014	P02051	P02087	P02120	P04240	P07409	P08535
A4GX73	O93348	P01953	P01985	P02015	P02052	P02088	P02121	P04241	P07410	P08849
A6YH87	O93349	P01954	P01986	P02016	P02053	P02089	P02122	P04242	P07411	P08850
A7UAU9	O93351	P01955	P01987	P02017	P02054	P02090	P02123	P04244	P07412	P08851
A9JSP7	O96457	P01956	P01988	P02018	P02055	P02091	P02124	P04245	P07413	P08852
A9XDF6	P01923	P01957	P01989	P02019	P02057	P02092	P02125	P04246	P07414	P08853
B0BL34	P01924	P01958	P01990	P02020	P02058	P02093	P02126	P04252	P07415	P09105
B0BL35	P01926	P01959	P01991	P02021	P02059	P02094	P02127	P04346	P07417	P09420
B1H216	P01928	P01960	P01992	P02022	P02060	P02095	P02128	P04442	P07419	P09421
B1Q450	P01929	P01961	P01993	P02024	P02061	P02097	P02129	P04443	P07421	P09422
B2BP38	P01930	P01962	P01994	P02025	P02062	P02099	P02130	P04444	P07425	P09423
B2ZUE0	P01932	P01963	P01995	P02026	P02064	P02100	P02131	P06148	P07428	P09839
O02004	P01933	P01964	P01996	P02028	P02065	P02101	P02132	P06467	P07429	P09840
O02480	P01934	P01965	P01997	P02029	P02066	P02102	P02133	P06635	P07430	P09904
O04985	P01935	P01966	P01998	P02030	P02067	P02103	P02134	P06636	P07431	P09905
O04986	P01936	P01967	P01999	P02031	P02070	P02104	P02135	P06637	P07432	P09906
O09232	P01937	P01968	P02000	P02032	P02072	P02105	P02136	P06638	P07433	P09907
O12985	P01938	P01969	P02001	P02033	P02073	P02106	P02137	P06639	P07803	P09908
O13077	P01939	P01971	P02002	P02035	P02074	P02107	P02138	P06642	P08054	P09909
O13078	P01940	P01972	P02003	P02036	P02075	P02108	P02139	P06643	P08223	P0C0U6
O13163	P01941	P01973	P02004	P02038	P02076	P02109	P02140	P07034	P08224	P0C0U7
O13164	P01942	P01975	P02005	P02039	P02077	P02110	P02141	P07035	P08225	P0C0U8

**Table 3 pone-0009550-t003:** SwissProt/UniProt Accession Numbers for Hemoglobin dataset – Part 2.

P0C237	P14524	P20018	P41327	P67822	P68194	P83132	Q27940	Q6AW44	Q8AXX7	Q9TSN9
P0C238	P14525	P20019	P41328	P67823	P68222	P83133	Q28220	Q6AW45	Q8AYL9	Q9TVA3
P0C239	P14526	P20243	P41329	P67824	P68223	P83134	Q28221	Q6B0K9	Q8AYM0	Q9U6L2
P0C240	P14527	P20244	P41330	P68011	P68224	P83135	Q28338	Q6BBJ0	Q8AYM1	Q9U6L6
P10057	P15162	P20245	P41331	P68012	P68225	P83270	Q28356	Q6BBJ2	Q8BPF4	Q9XSE9
P10058	P15163	P20246	P41332	P68013	P68226	P83271	Q28496	Q6BBK1	Q8BYM1	Q9XSK1
P10059	P15164	P20247	P45718	P68014	P68227	P83272	Q28507	Q6H1U7	Q8GV40	Q9XSN2
P10060	P15165	P20854	P45719	P68015	P68228	P83273	Q28775	Q6IBF6	Q8GV41	Q9XSN3
P10061	P15166	P20855	P45720	P68016	P68229	P83478	Q28779	Q6LDH0	Q8GV42	Q9XTL1
P10062	P15448	P21197	P45721	P68017	P68230	P83479	Q28931	Q6LDH1	Q8HY34	Q9XTM2
P10777	P15449	P21198	P45722	P68018	P68231	P83611	Q28932	Q6QDC2	Q8QG65	Q9Y0D5
P10778	P15469	P21199	P51438	P68019	P68232	P83612	Q29415	Q6R7N2	Q90485	Q9Y0E6
P10780	P16309	P21200	P51440	P68020	P68234	P83613	Q2KPA3	Q6S9E2	Q90486	Q9YGW1
P10781	P16417	P21201	P51441	P68021	P68235	P83614	Q2KPA4	Q6T497	Q90487	Q9YGW2
P10782	P16418	P21379	P51442	P68022	P68236	P83623	Q2MHE0	Q6WN20	Q90ZM4	
P10783	P17689	P21380	P51443	P68023	P68237	P83624	Q2PAD4	Q6WN21	Q91473	
P10784	P18435	P21667	P51465	P68024	P68238	P83625	Q3C1F3	Q6WN22	Q941P9	
P10785	P18436	P21668	P55267	P68025	P68239	P84203	Q3C1F4	Q6WN25	Q941Q1	
P10786	P18707	P21766	P56250	P68026	P68240	P84204	Q3MQ26	Q6WN26	Q946U7	
P10883	P18969	P21767	P56251	P68027	P68256	P84205	Q3S3T0	Q6WN27	Q947C5	
P10885	P18970	P21768	P56285	P68028	P68257	P84206	Q3TUN7	Q6WN28	Q94FG6	
P10892	P18971	P21871	P56691	P68029	P68258	P84216	Q3U0A6	Q6WN29	Q94FT7	
P10893	P18972	P22740	P56692	P68030	P68871	P84217	Q3Y9L5	Q6Y239	Q94FT8	
P11025	P18973	P22741	P60523	P68031	P68872	P84479	Q3Y9L6	Q6Y257	Q95190	
P11251	P18974	P22742	P60524	P68044	P68873	P84604	Q42665	Q760P9	Q95238	
P11342	P18975	P22743	P60525	P68045	P68944	P84609	Q42831	Q760Q0	Q95NK8	
P11517	P18976	P23016	P60526	P68046	P68945	P84610	Q43306	Q760Q1	Q966U3	
P11748	P18977	P23017	P60529	P68047	P69891	P84611	Q45V69	Q760Q2	Q96FH6	
P11749	P18978	P23018	P60530	P68048	P69892	P84652	Q45V70	Q78PA4	Q98905	
P11750	P18981	P23019	P61772	P68049	P69905	P84653	Q45XH3	Q7JFN6	Q98TS0	
P11751	P18982	P23020	P61773	P68050	P69906	P84790	Q45XH5	Q7JFR7	Q9AWA9	
P11752	P18983	P23600	P61774	P68051	P69907	P84791	Q45XH6	Q7LZB9	Q9CWS5	
P11753	P18984	P23601	P61775	P68052	P80043	P84792	Q45XH7	Q7LZC1	Q9CY06	
P11754	P18985	P23602	P61920	P68053	P80044	P85081	Q45XH8	Q7LZC2	Q9CY10	
P11755	P18986	P23740	P61921	P68054	P80216	P85082	Q45XI4	Q7LZC3	Q9CZK5	
P11756	P18987	P23741	P61947	P68055	P80270	Q03902	Q45XI5	Q7LZL6	Q9D0B2	
P11757	P18988	P24291	P61948	P68056	P80271	Q0PB48	Q45XI6	Q7LZM6	Q9DF25	
P11758	P18989	P24292	P62363	P68057	P80726	Q0PG38	Q45XI7	Q7LZM7	Q9FUD6	
P11896	P18990	P24589	P62387	P68058	P80727	Q0WSU5	Q45XI8	Q7LZM8	Q9FVL0	
P13273	P18993	P24659	P62741	P68059	P80945	Q0ZA50	Q45XI9	Q7M2Y4	Q9FY42	
P13274	P18994	P24660	P62742	P68060	P80946	Q10732	Q45XJ0	Q7M2Y5	Q9GJS7	
P13557	P18995	P26915	P63105	P68061	P81023	Q10733	Q4F6Z2	Q7M3B6	Q9GLX4	
P13558	P18996	P26916	P63106	P68062	P81024	Q17153	Q4VIX3	Q7M3B8	Q9I9I3	
P14259	P19002	P28780	P63107	P68063	P81042	Q17155	Q53I65	Q7M3C2	Q9M3U9	
P14260	P19014	P28781	P63108	P68064	P81043	Q17156	Q549D9	Q7M413	Q9M593	
P14261	P19015	P29623	P63109	P68065	P82111	Q17157	Q549G1	Q7M418	Q9M630	
P14387	P19016	P29624	P63110	P68068	P82112	Q17286	Q58L97	Q7M419	Q9PRL9	
P14388	P19645	P29625	P63111	P68069	P82113	Q1AGS4	Q5BLF6	Q7M421	Q9PVM1	
P14389	P19646	P29626	P63112	P68070	P82315	Q1AGS5	Q5GLZ6	Q7M422	Q9PVM2	
P14390	P19759	P29628	P67815	P68071	P82316	Q1AGS6	Q5GLZ7	Q7T1B0	Q9PVM3	
P14391	P19760	P30892	P67816	P68077	P82345	Q1AGS7	Q5I122	Q7Y079	Q9PVM4	
P14392	P19789	P30893	P67817	P68078	P82990	Q1AGS8	Q5KSB7	Q7Y1Y1	Q9PVU6	
P14520	P19831	P33499	P67818	P68079	P83114	Q1AGS9	Q5MD69	Q7ZT21	Q9TS34	
P14521	P19832	P41260	P67819	P68087	P83123	Q1W6G9	Q5RM02	Q803Z5	Q9TS35	
P14522	P19885	P41261	P67820	P68168	P83124	Q26505	Q5XLE5	Q862A7	Q9TSN7	
P14523	P19886	P41262	P67821	P68169	P83131	Q27126	Q67XG0	Q86G74	Q9TSN8	

**Table 4:** SwissProt/UniProt Accession Numbers for Myoglobin dataset.

O77003	P02159	P02174	P02190	P02205	P14393	P51535	P68086	Q01966	Q701N9	Q9DEP1
P02144	P02160	P02177	P02191	P02206	P14396	P51537	P68189	Q03459	Q76G09	Q9DGI8
P02145	P02161	P02178	P02192	P02210	P14397	P56208	P68190	Q0KIY0	Q7LZM1	Q9DGI9
P02147	P02163	P02179	P02193	P02211	P14398	P62734	P68276	Q0KIY1	Q7LZM2	Q9DGJ0
P02148	P02164	P02180	P02194	P02214	P14399	P62735	P68277	Q0KIY2	Q7LZM3	Q9DGJ1
P02150	P02165	P02181	P02196	P02215	P15160	P63113	P68278	Q0KIY3	Q7LZM4	Q9DGJ2
P02151	P02166	P02182	P02197	P04247	P17724	P63114	P68279	Q0KIY5	Q7LZM5	Q9QZ76
P02152	P02167	P02183	P02199	P04248	P20856	P68080	P80721	Q0KIY7	Q7M416	
P02153	P02168	P02184	P02200	P04249	P29287	P68081	P80722	Q0KIY9	Q7M424	
P02154	P02169	P02185	P02201	P04250	P30562	P68082	P83682	Q2MJN4	Q7T044	
P02155	P02170	P02186	P02202	P09965	P31331	P68083	P84997	Q6I7B0	Q9DEN8	
P02156	P02171	P02187	P02203	P0C227	P32428	P68084	P85077	Q6PL31	Q9DEN9	
P02157	P02173	P02189	P02204	P11343	P49672	P68085	P87497	Q6VN46	Q9DEP0	

### Visualization of the Feature Vectors

The feature vector is high dimensional. PCA is a popular method of reducing the number of variables in a vector. This method provides a linear transformation of the variables of the feature vector into a new set of uncorrelated variables. In so doing, it captures the dominant variations in the data set. The first two principal components contain more information than any other pair of linearly constructed variables and thus are used in our analysis [6]. This allows us to easily visualize the key elements of the data without loss of much information.

An alternative method for visualizing the patterns emerging from the feature vector method of analysis is to create a dendrogram using agglomerative hierarchical clustering. We use agglomerative hierarchical clustering with complete linkage [7] to provide a more detailed view of the data, and of the relationships between groups within the data. In order to demonstrate the utility and flexibility of the feature vector method, we provide one dendrogram as part of the PKC analysis.

### Identification of Protein Sub-Types

**Figure 2:**
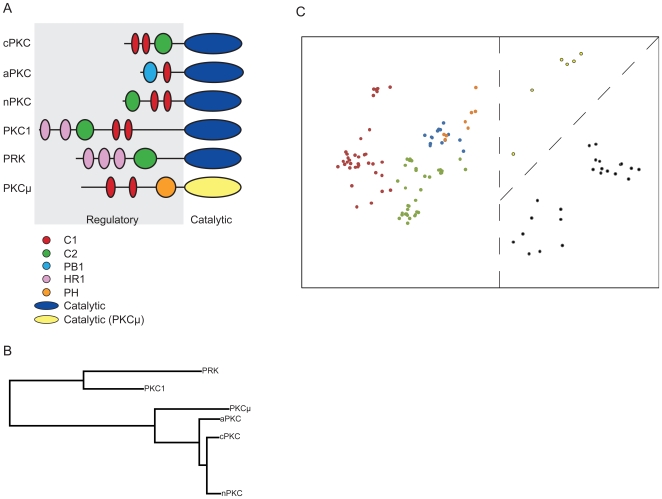
Analysis of the Protein Kinase C (PKC) family using the feature vector method. (A) Structural architecture types of PKCs used in analysis, showing regulatory domains – C1, C2, PB1, HR1 and PH – and catalytic domains. cPKC – conventional PKCs, aPKC – atypical PKCs, nPKC – novel PKCs, PKC1 – fungal PKC1s, PRK – PKC-related kinases, PKCμ – PKCμ-like PKCs; (B) agglomerative hierarchical clustering dendrogram of PKC feature vectors for structural architecture types; (C) principle component analysis (PCA) of PKC feature vectors, coded by known architectural type of proteins – red dots: cPKCs; blue dots: aPKCs; green dots: nPKCs; black dots: PKC1s; orange dots: PKCμ; yellow dots: PRKs; dashed lines indicate dividing surfaces for identifying major clusters in the data set.

Kinases are proteins which modify other proteins by phosphorylation, the covalent addition of phosphate groups [8]. The PKC family is a large multigene family of serine/threonine kinases [8]. Six main groups of PKCs can be identified by domain architecture: conventional, novel, atypical, PKCμ-like, fungal PKC1, and PKC-related kinases [9]. The first three of these groups can be further categorized into subtypes [9]. In general, the PKC domain architecture (fig. 2A) consists of a regulatory region and a catalytic domain [10]. The regulatory region contains several functional domains of varying types [10]. True PKCs are classified as conventional, novel or atypical based on the functional domains present in the regulatory regions of the PKCs (fig. 2A). Briefly, conventional PKCs contain subtypes α, βI, βII and γ; novel PKCs contain subtypes θ, ε, δ and η; and atypical PKCs contain λ\ι and ζ [9]. The catalytic domain is more conserved and more commonly used for differentiating between families of protein kinases [11]. However, it is also useful in characterizing the PKCμ-like kinases, which contain markedly different catalytic regions from the rest of the protein kinase C members [12].

The dendrogram created by agglomerative hierarchical clustering of the PKC feature vectors (fig. 2B) successfully recreates the phylogenetic relationships between PKC architectural types and highlights the degree of difference between PKC-related kinases, PKC1s and other PKCs.

By running a PCA on the feature vector values for each PKC protein, we were able to quickly visualize the six architecture types of PKCs in our dataset. Figure 2C shows the PCA output for PKCs. A dashed line marking a clear dividing surface is added to this figure to demonstrate divisions in the data that warrant further analysis. Fungal PKC1s are clearly separated from other PKCs and can be identified as an important, distinct grouping (fig. 2C). In addition, PKC-related kinases and true PKCs are located in distinct clusters (fig. 2C). Finally, conventional PKCs and novel PKCs are resolved into distinct clusters (fig. 2C).

**Figure 3:**
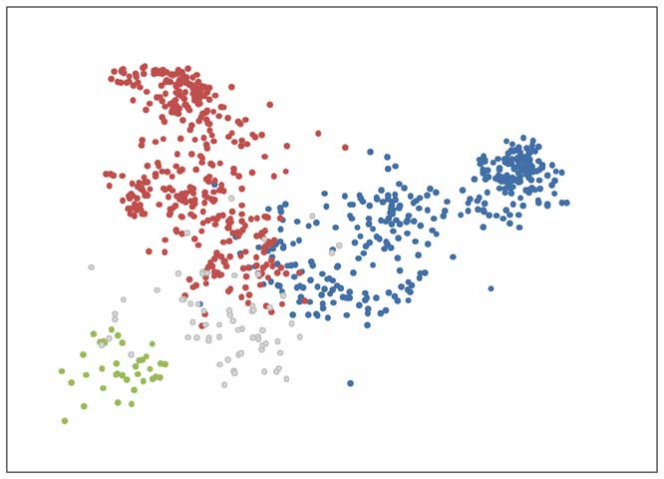
Analysis of hemoglobin proteins using the feature vector method. PCA results of hemoglobin feature vectors, coded by known protein type – red dots: beta-chain hemoglobins; blue dots: alpha-chain hemoglobins, including fetal alpha-type proteins; green dots: leghaemoglobins; grey dots: other hemoglobins.

A similar identification of hemoglobin structure was also possible (fig. 3). Hemoglobins are large proteins which function in oxygen transport [13]. Each hemoglobin molecule contains 2 alpha chains and 2 beta chains, subunits which are identifiable by structural characteristics [13]. Alpha hemoglobins lack a specific alpha-helix, the D helix, that is present in beta hemoglobins [14]. Many organisms have several distinct hemoglobins, an adult form and embryonic or fetal forms created by combining different alpha and beta hemoglobin units [13]. There are more types of embryonic and fetal alpha hemoglobins than beta hemoglobins, and thus alpha hemoglobins are presumed evolutionarily older [13]. In protein databases, alpha and beta chain hemoglobins belonging to a given species are typically recorded in separate entries and so are separate in our data set also.

Using the feature vector method, the PCA identifies the difference between alpha chain hemoglobins, beta chain hemoglobins and leghaemoglobins, a type of monomeric hemoglobin chain found in plants [15], as the main features of the protein set (fig. 3). A range of other types of hemoglobins occur in the dataset in small numbers, but are not well resolved into distinct clusters due to their rarity in the data (see below). These proteins include the non-symbiotic plant hemoglobins, also called truncated or 2-on-2 hemoglobins [16]; the lamprey/hagfish hemoglobins [17]; bacterial hemoglobins; and erythrocruorins, which are large extracellular hemoglobins found in annelid worms and arthropods [18].

**Figure 4:**
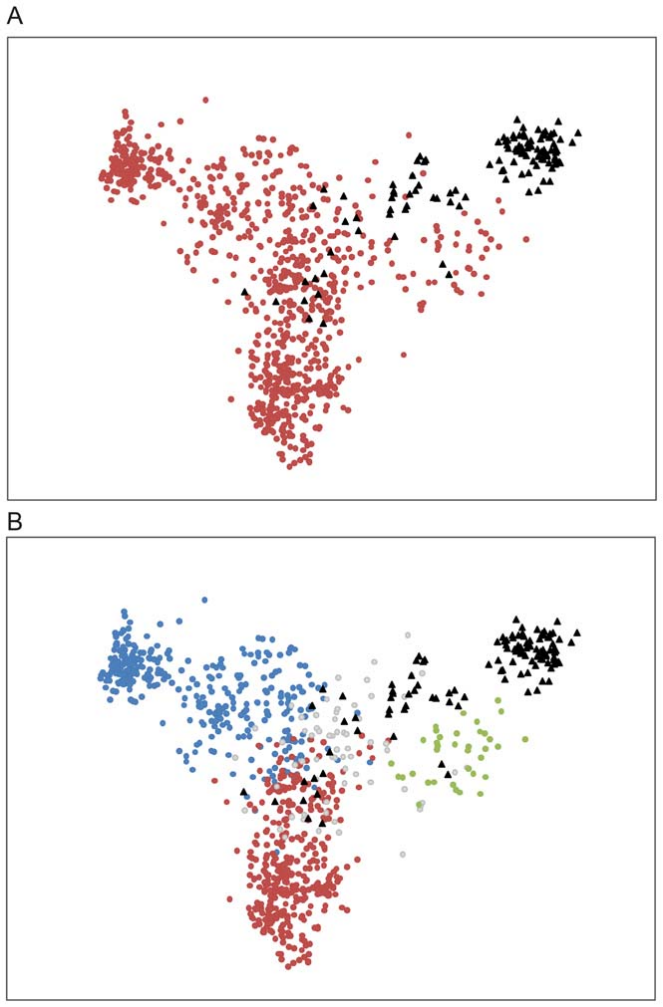
Identification of protein type as myoglobin and hemoglobin by feature vector analysis. (A) PCA results of hemoglobin and myoglobin feature vectors, coded by known protein family – red dots: all hemoglobins; black triangles: myoglobins; (B) PCA results of hemoglobin and myoglobin feature vectors, coded by known protein type with hemoglobins identified by subtype – red dots: beta-chain hemoglobins; blue dots: alpha-chain hemoglobins, including fetal alpha-type proteins; green dots: leghaemoglobins; grey dots: other hemoglobins; black triangles: myoglobins.

The feature vector method is able to store large amounts of information about the proteins in the dataset. When the 150 myoglobins are added to the hemoglobin dataset, the feature vector is able to distinguish these two types of proteins (fig. 4A), while retaining information about structural relationships within the hemoglobin family (fig. 4B). Myoglobins are single chain hemoproteins and share a common ancestor with hemoglobins, more than 500 million years ago [19]. Structurally, myoglobins are similar to leghaemoglobins, but functionally these proteins are quite different, with leghaemoglobins having significantly higher oxygen affinity and a broad range of functions within plant nodules [15], [19], [20]. The feature vector method, combined with PCA for visualization, clearly separates the majority of myoglobins from hemoglobins (fig. 4A), while preserving the ability to identify structural relationships between alpha and beta hemoglobins and leghaemoglobins (fig. 4B).

**Figure 5:**
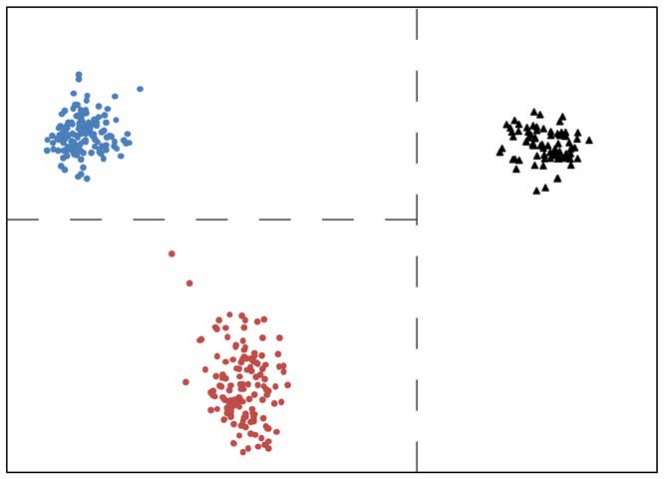
Use of the feature vector method allows unequivocal protein identification when data is limited to large, well-defined protein types. PCA results of adult alpha and adult beta mammalian hemoglobins and mammalian myoglobin feature vectors, coded by known protein type to demonstrate the ability of the feature vector to produce perfect separation of types – dashed lines indicate dividing surfaces for identifying clusters in the data; red dots: beta-chain mammalian hemoglobins; blue dots: alpha-chain mammalian hemoglobins, excluding fetal proteins; black triangles: mammalian myoglobins.

When analyzing a large and varied protein dataset, some protein types may occur infrequently. These rare protein types are more difficult to cluster using PCA due to the limited amount of information available to the algorithm. As a result, these rare proteins cluster near the center of the PCA output, creating ‘noise’ in the analysis (fig. 3). By limiting the hemoglobin dataset to only adult alpha and beta chain mammalian hemoglobins and mammalian myoglobins, the most frequent protein types present in the dataset, the ability of the feature vector method to create clear separation between different groups of proteins is readily apparent (fig. 5). As in previous figures, dashed lines indicating decision surfaces are used to highlight clusters warranting further analysis.

## Discussion

The feature vector method described here is intended to measure the distance between protein sequences in a way that makes numerical comparisons easy and allows identification of similarity within large numbers of proteins that are not too distantly related. Using PKCs and hemoproteins as examples, we demonstrated the effectiveness of this method. When groups are completely distinct, perfect separation can be achieved; where there are gradual changes in the sequences of proteins, the feature vector performs well in conjunction with principal components analysis. Importantly, this method does not attempt to characterize differences as functional or non-functional, nor does it seek to identify key single point mutations. Rather, the goal is to provide a rapid understanding of the patterns of relatedness in large datasets of protein sequences.

Although protein kinase C was one of the first protein kinases discovered [21], categorizing members of this family is particularly challenging [22], [23]. Our method successfully reproduces the traditional classification of PKCs and clusters family members on the basis of these classifications [24]. Previous work analyzing relationships among multiple PKCs or among the larger kinase superfamily has been limited by the maximum dataset size [23], [24], [25], in a way that our method is not. The statistical feature vector method is particularly useful as a simple way of identifying subgroups in non-mammalian PKCs, an area where little is known. In the future, more detailed visualization techniques may suggest new relationships which could be explored experimentally.

Mammalian hemoglobins are also well understood, in terms of classification [16]. However, research is increasingly identifying hemoglobin-like proteins outside of mammals, including bacterial hemoglobins and non-symbiotic hemoglobins in plants [16]. As the number of hemoglobins identified in these organisms increases, the feature vector method will provide a simple tool for identifying structural groupings within these proteins.

The feature vector method provides one of the most definitive ways of classifying various types of proteins. This method provides an advantage over other classification programs in ease of use and, unlike other methods, the feature vector is not constrained to a single protein family or superfamily [23]. We have shown the usefulness of this method in PKCs and hemoproteins, and we anticipate that it will perform equally well when applied to other protein families providing a simple, rapid tool for sorting through the increasingly large datasets of proteins now available to researchers.

In the future, the utility of this method can be increased by applying new, and more specific, visualization tools to the analysis of the feature vector output, such as K-means, agglomerative hierarchical clustering, artificial neural networks and self-organizing maps. For a given data set, the patterns of variation in sequences can be learned by neural networks, or other methods, to provide a more accurate classification or clustering than can be achieved with less flexible methods like principal components analysis.

## Methods

### Datasets

We used three online protein sequence databases to create our protein datasets: Uniprot KB, UniprotKB/Swissprot, and NCBI Entrez-Protein. UniprotKB (www.uniprot.org) is an online repository of protein sequences; UniprotKB/Swissprot (http://ca.expasy.org/sprot/) builds upon this repository through annotation of protein sequences. Information available in UniprotKB/Swissprot includes citations for related publications, species name, protein family, domain structure and detail on protein variants and structure. NCBI Entrez-Protein (http://www.ncbi.nlm.nih.gov/protein/) is an online protein sequence database curated by the National Center for Biotechnology Information (NCBI).

The protein kinase C dataset of 127 protein sequences was downloaded from the NCBI Entrez-Protein and UniProtKB/SwissProt databases. The hemoglobin and myoglobin datasets, of 904 and 150 protein sequences respectively, were downloaded from the UniProtKB database. In order to ensure that sequences were not fragments or labeled incorrectly by protein family, sequences were analyzed using the SMART domain recognition software on the UniProtKB website. In addition, for all sequences the family classification was confirmed and the subfamily classification was assigned based on peer-reviewed journal articles which were obtained through the SwissProt database reference listings and based on notations on the UniProtKB entries where detailed information from articles was not available.

## Supporting Information

Supplement S1 Sample Parameter Calculations. This file works through the calculations of the parameters for Alanine in a short, hypothetical protein, and demonstrates the construction of the feature vector for this protein.
(0.05 MB PDF)

Sample Code S1 Feature vectors computation in C++. Computation of the feature vectors for a protein data set in C++.
(0.01 MB TXT)

Sample Code S2 Feature vector computation in Mathematica. Computation of the feature vector for a single protein in Mathematica.
(0.00 MB TXT)

Sample Code S3 PCA code for Matlab. Principle component analysis code for Matlab.
(0.00 MB TXT)

Table S1 Protein Kinase C Feature Vectors. This file contains the set of all feature vectors for the PKC proteins in our dataset.
(0.16 MB XLS)

Table S2 Hemoglobin Feature Vectors. This file provides the set of all feature vectors for the Hemoglobins in our dataset.
(1.03 MB XLS)

Table S3 Myoglobin Feature Vectors. This file provides the set of all feature vectors for the Myoglobins in our dataset.
(0.17 MB XLS)

Data Set S1 Protein datasets. Accession numbers and taxonomic information for Protein Kinase C (PKC), Hemoglobin and Myoglobin dataset. Each protein dataset is provided as a separate worksheet.
(0.16 MB XLS)
