# Supplementary material for: A Rapid Method for Characterization of Protein Relatedness Using Feature Vectors
Source: PLoS One. 2010 Mar 5;5(3):e9550. doi: 10.1371/journal.pone.0009550 (PMC2832692; doi:10.1371/journal.pone.0009550)
Supplement: Supplement S1 — Sample Parameter Calculations. This file works through the calculations of the parameters for Alanine in a short, hypothetical protein, and demonstrates the construction of the feature vector for this protein. (0.05 MB PDF) [file pone.0009550.s001.pdf]

A Sample Calculation of Parameters

The necessary calculations for computing parameters associated with a particular type of amino acid are straightforward. Using the sample protein,

ARRRAARA,

we will demonstrate the calculations for the parameters associated with alanine (A).

The length of the protein is $N = 8$. The list of positions are: $\{1, 5, 6, 8\}$. Therefore, $n_A = 4$.

Type I

measure

$$p_A = \frac{n_A}{N} = \frac{4}{8}$$

theoretical mean

$$\pi_A = \frac{4}{61}$$

This value is due to four codons coding for alanine out of 61 possible coding codons. This is used as an estimate of the probability of an amino acid occurring in a particular protein. Using better estimates based on specialized knowledge of the proteins being classified would improve the effectiveness of the method, and is recommended.

theoretical variance

$$\frac{\pi_A (1 - \pi_A)}{N} = \frac{\frac{4}{61}\left(1 - \frac{4}{61}\right)}{8} = \frac{57}{7442}$$

parameter value

$$\frac{\frac{4}{8} - \frac{4}{61}}{\sqrt{\frac{57}{7442}}} \approx 4.9639$$

Type II

measure

$$\bar{t} = \frac{1}{n_A}\sum_{i=1}^{n_A} t_i = \frac{(1+5+6+8)}{4} = 5$$

theoretical mean

$$\frac{N+1}{2} = \frac{8+1}{2} = \frac{9}{2}$$

theoretical variance

$$\frac{(N+1)(N-n_A)}{12n_A} = \frac{(8+1)(8-4)}{12\cdot 4} = \frac{3}{4}$$

parameter value

$$\frac{5-\frac{9}{2}}{\sqrt{\frac{3}{4}}} \approx 0.57735$$

Type III

measure

$$\frac{N-1}{N}\cdot\frac{1}{n_A-1}\sum_{i=1}^{n_A}(t_i-\bar{t}) =$$

$$\frac{7}{8}\cdot\frac{(1-5)^2+(5-5)^2+(6-5)^2+(8-5)^2}{3} = \frac{91}{12}$$

theoretical mean

$$\frac{N^2-1}{12} = \frac{8^2-1}{2} = \frac{63}{2}$$

theoretical variance

$$\frac{(N-n_A)(N-1)^2(N+1)(2n_AN+3N+3n_A+3)}{360n_A(n_A-1)N} =$$

$$\frac{(8-4)(8-1)^2(8+1)(2\cdot 4\cdot 8+3\cdot 8+3\cdot 4+3)}{360\cdot 4\cdot (4-1)\cdot 8} = \frac{5047}{960}$$

parameter value

$$\frac{\frac{91}{12} - \frac{63}{12}}{\sqrt{\frac{5047}{960}}} \approx 1.01764$$

Feature Vector

These three parameters are computed for the remaining nineteen amino acids. Any parameter that is undefined, due to a zero denominator in one of the required calculations (that is, the amino acid does not occur in the protein), is set to zero. The feature vector is constructed as a vector of all parameters for all amino acids, in alphabetical order by name (rather than by symbol).

In this case,

{4.9639, 0.57735, 1.01764, ... }.
